# Characterization, in Vitro and in Vivo Evaluation of Naringenin-Hydroxypropyl-β-Cyclodextrin Inclusion for Pulmonary Delivery

**DOI:** 10.3390/molecules25030554

**Published:** 2020-01-28

**Authors:** Minyi Guan, Rui Shi, Yuying Zheng, Xuan Zeng, Weiyang Fan, Yonggang Wang, Weiwei Su

**Affiliations:** Guangdong Engineering & Technology Research Center for Quality and Efficacy Reevaluation of Post-Market Traditional Chinese Medicine, Guangdong Key Laboratory of Plant Resources, School of Life Sciences, Sun Yat-sen University, No. 135, Xingang Xi Road, Guangzhou 510275, China; mandyguan723@163.com (M.G.); ruishi900930@gmail.com (R.S.); vicky_0224@126.com (Y.Z.); zengx6@mail2.sysu.edu.cn (X.Z.); fanwy5@mail2.sysu.edu.cn (W.F.); wangyg@mail.sysu.edu.cn (Y.W.)

**Keywords:** naringenin, hydroxypropyl-β-cyclodextrin, pulmonary delivery, NMR, permeability, pharmacokinetic

## Abstract

Naringenin, a flavonoid compound which exists abundantly in *Citrus* fruits, is proven to possess excellent antitussive and expectorant effects. However, the clinical applications of naringenin are restricted by its poor solubility and low local concentration by oral administration. The aim of the present study is to prepare a naringenin-hydroxypropyl-β-cyclodextrin (naringenin-HPβCD) inclusion as an inhalation solution for pulmonary delivery. The naringenin-HPβCD inclusion was characterized by phase solubility study, XRD, differential scanning calorimetry (DSC), proton nuclear magnetic resonance (^1^HNMR), and two-dimensional rotating frame Overhauser effect spectroscopy (2D ROESY). The in vitro permeability of the inclusion was evaluated on Calu-3 cells and the pharmacokinetic profile of pulmonary delivery was investigated in Sprague-Dawley (SD) rats. Based on the linear model of phase solubility study, the relationship between naringenin and HPβCD was identified as A^L^ type with a 1:1 stoichiometry. XRD, DSC, and NMR studies indicated that the entire naringenin molecule is encapsulated into the cavity of HPβCD. HPβCD could increase the concentration of naringenin in the epithelium-lining fluid (ELF) of Calu-3 cells and act as a sustained release system for naringenin. The pharmacokinetic profile of naringenin-HPβCD inclusion showed rapid response and higher local concentration by pulmonary delivery. In conclusion, pulmonary delivery of naringenin-HPβCD inclusion is a promising formulation strategy, which could provide a new possibility for the clinical application of naringenin.

## 1. Introduction

**Figure 1:**
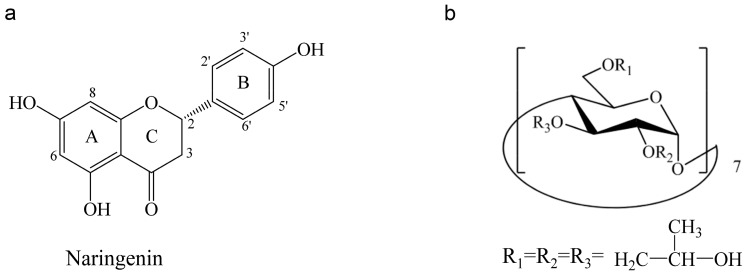
(**a**) The chemical structure of naringenin; (**b**) hydroxypropyl-β-cyclodextrin (HPβCD).

Naringenin (5,7,4′-trihydroxyflavanone, Figure 1a) is a flavanone found abundantly in *Citrus* fruits, such as grapefruits, oranges and pummelos, and it is also the active ingredient of many Chinese herbal medicines [1]. As a polyphenolic compound, naringenin possesses diverse pharmacological activities such as anti-oxidant, anti-inflammatory, anti-atherogenic, and hepatoprotective [2]. In recent years, naringenin and its glucoside have been proven to exhibited excellent expectorant and antitussive effects in various in vitro and in vivo models, indicating that naringenin is a promising drug for the treatment of respiratory diseases [3,4,5,6,7,8,9,10,11].

Regretfully, the clinical applications of naringenin are limited by its poor solubility and bioavailability. A large number of formulation strategies were employed to solve these problems, including inclusion complexes, self-nanoemulsifying drug delivery system, solid dispersions, and nanoparticles [1,2,12,13,14,15]. However, these formulations were designed for oral administration, which has limited improvement of bioavailability due to the first pass effect, and tissue distribution study showed that most naringenin was concentrated in the gastrointestinal tract, while a little naringenin could reach the lungs [16,17,18]. Therefore, to achieve better therapeutic efficiency for respiratory diseases, it is necessary to develop a new delivery route for naringenin.

Benefits from the large surface area (~100 m^2^) and the thin absorption membrane (~0.1–0.2 µm) of the lung, delivering drugs as an aerosol via pulmonary route allows rapid absorption, high local concentration, and systemic side-effect reduction of the drugs, which becomes a promising administration route in the treatment of respiratory diseases, such as asthma, chronic pulmonary infections, fibrosis or lung cancer [19]. It is noteworthy that prior to absorption, the drug should dissolve in the very thin pulmonary surface liquid [20], which is a challenge for naringenin due to its poor solubility and dissolution rate.

Cyclodextrins are cyclic oligosaccharides composed of covalently linked glucopyranose rings. They can provide a solution to increase the solubility of hydrophobic drugs by forming water-soluble inclusions, which encapsulate the entire or part of drugs in the hydrophobic cavities of cyclodextrins [21]. Hydroxypropyl-β-cyclodextrin (HPβCD) (Figure 1b) is a chemically modified derivate of cyclodextrins with much higher solubility in water, and can be used safely as a complexing and solubilizing excipient in various administration routes of drugs. A number of studies reports on the applications of HPβCD solution for pulmonary delivery, in which the HPβCD solution shows a compatible range of droplet size with pulmonary deposition, sustain-release characteristic in drug absorption, and non-toxic in short-term exposure [22,23,24].

In the present study, a naringenin-HPβCD inclusion was prepared with the aim of increasing the solubility and local concentration of naringenin. The solubilizing ability and complexation efficiency of HPβCD with naringenin were demonstrated by the phase solubility study. The complexation mode of the inclusion was illustrated by nuclear magnetic resonance (NMR) studies. Calu-3 cells were employed as broncho-alveolar epithelial cell model to evaluate the in vitro performance of naringenin inclusion and the in vivo pharmacokinetic profile was investigated in SD rats.

## 2. Results and Discussion

### 2.1. Phase Solubility Study

**Figure 2:**
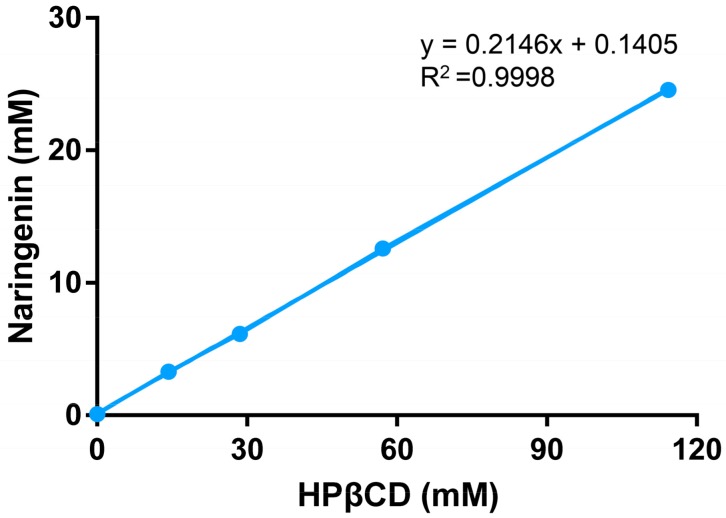
The phase solubility diagram of naringenin in the presence of HPβCD over a concentration range from 0 to 114.29 mM (mean ± SD, *n* = 3).

The phase solubility diagram for naringenin in HPβCD system is shown in Figure 2. The solubility of naringenin increased linearly (37 to 273 times) as a function of HPβCD concentration over the studied concentration range, which could be classified as A^L^ type according to Higuchi and Connors [21]. The slope value was less than one, indicating the formation of a 1:1 stoichiometry inclusion between naringenin and HPβCD. The stability constant K_s_ is introduced to present the solubilizing efficiency of cyclodextrins for a drug, and the complexation efficiency is used to describe the ratio between cyclodextrin inclusions and free cyclodextrins [25]. In this work, the K_s_ and complexation efficiency (CE) values were 2732.37(M^−1^) and 0.2732, respectively. The molar ratio of naringenin to HPβCD was about 1:5, suggesting that 1 out of every 5 HPβCD molecules formed an inclusion with naringenin.

### 2.2. Powder X-ray Diffraction (XRD)

**Figure 3:**
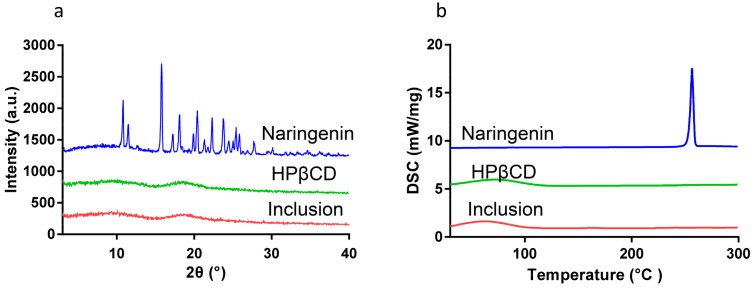
(**a**) XRD profile and (**b**) DSC thermogram of naringenin, HPβCD, and the inclusion.

The XRD spectra of naringenin, HPβCD, and their inclusion are shown in Figure 3. Naringenin exhibited its crystalline property with strongly sharp peaks in the range of 2θ from 3° to 40°, while HPβCD displayed an amorphous property with no distinct peaks observed in the corresponding range. There was also no sharp peak shown in the XRD spectrum of naringenin-HPβCD inclusion, suggesting that naringenin was encapsulated in HPβCD.

### 2.3. Differential Scanning Calorimetry (DSC)

The thermogram of naringenin, HPβCD, and their inclusion are shown in Figure 3b. The DSC curve of naringenin showed an endothermic peak at 256 °C, while the DSC curve of HPβCD contained an endothermic peak at 72 °C. However, in the DSC curve of naringenin-HPβCD inclusion, the endothermic peaks at 255 °C corresponding to naringenin disappeared, and the inclusion exhibited the same endothermic peaks at 72 °C as the one of HPβCD, revealing the formation of the naringenin-HPβCD inclusion.

The Fourier transform infrared spectroscopy (FTIR) was also conducted to confirm the formation of naringenin-HPβCD inclusion. The results are shown in the Supplementary Materials.

### 2.4. Proton Nuclear Magnetic Resonance ^(1^HNMR) and Two Dimensional Rotating frame Overhauser Effect Spectroscopy (2D-ROESY) Studies

**Figure 4:**
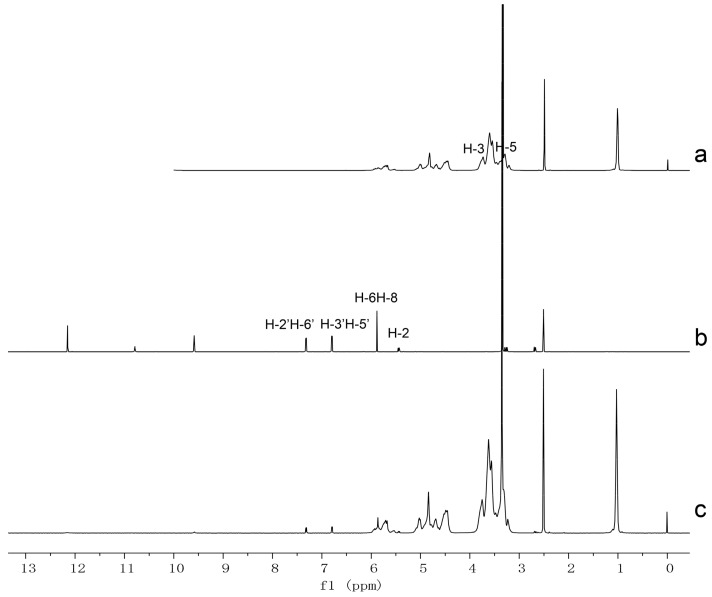
Proton nuclear magnetic resonance (^1^HNMR) spectra of (**a**) HPβCD, (**b**) naringenin, and (**c**) naringenin-HPβCD inclusion.

^1^HNMR technique is an effective tool for predicting the complexation mode of cyclodextrin inclusion by detecting the chemical shift changes of host and guest molecules (Δδ, Δδ = δinclusion − δfree) [26]. In the present study, the ^1^HNMR signals of naringenin, HPβCD, and naringenin-HPβCD inclusion were compared. The ^1^HNMR spectra and selected chemical shifts of naringenin and HPβCD are shown in Figure 4 and Table 1. As presented in Figure 4, the intensity of signals of naringenin in inclusion was low due to the small percentage in the inclusion. H-6 or H-8, H-2, H-3′ or H-5′, H-2′ or H-6′ are protons of ring A to C of naringenin; all these protons show chemical shift changes after the formation of inclusion, indicating that the entire naringenin molecule is inserted into the cavity of HPβCD. Among the chemical shift changes of these protons, H-6 or H-8, the protons of ring A of naringenin (Δδ = −0.021) exhibit more significant changes than those of H-3′ or H-5′ and H-2′ or H-6′ (Δδ = −0.004), the protons of ring C of naringenin. Thus, it can be deduced that ring A of naringenin is located at a deeper position in the cavity of HPβCD. In terms of the chemical shift changes of HPβCD, it is noteworthy that H-3 and H-5 protons are positioned in the interior of HPβCD, while H-3 protons are near the wide side, H-5 protons are near the narrow side [27]. Therefore, our attention focused on H-3 and H-5 protons. As described in Table 1, the chemical shift changes of H-3 protons were more significant than those of H-5 protons, indicating that the naringenin molecule penetrated into the HPβCD cavity from the wide side. Based on these results, together with the 1:1 stoichiometry of naringenin-HPβCD inclusion, we proposed that the entire naringenin molecule entered the HPβCD cavity from the wide side by orienting ring A of naringenin to the narrow side.

**Figure 5:**
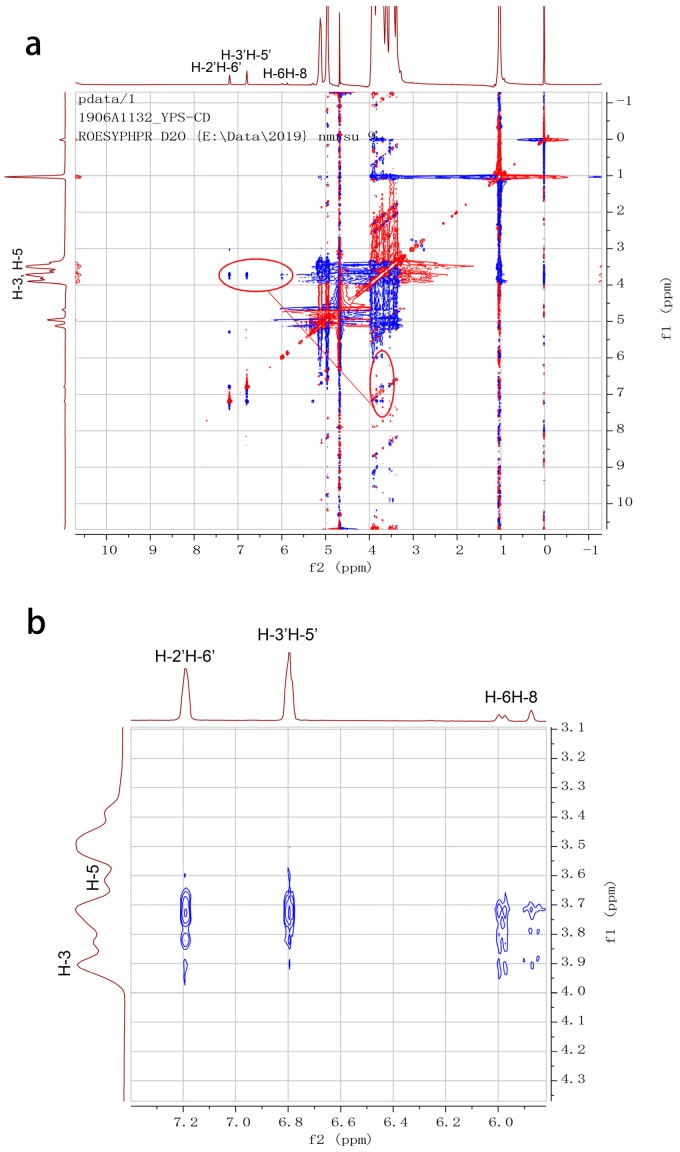
Two-dimensional rotating frame Overhauser effect spectroscopy (2D ROESY) spectrum of (**a**) naringenin-HPβCD inclusion and (**b**) partial expansion spectrum.

**Figure 6:**
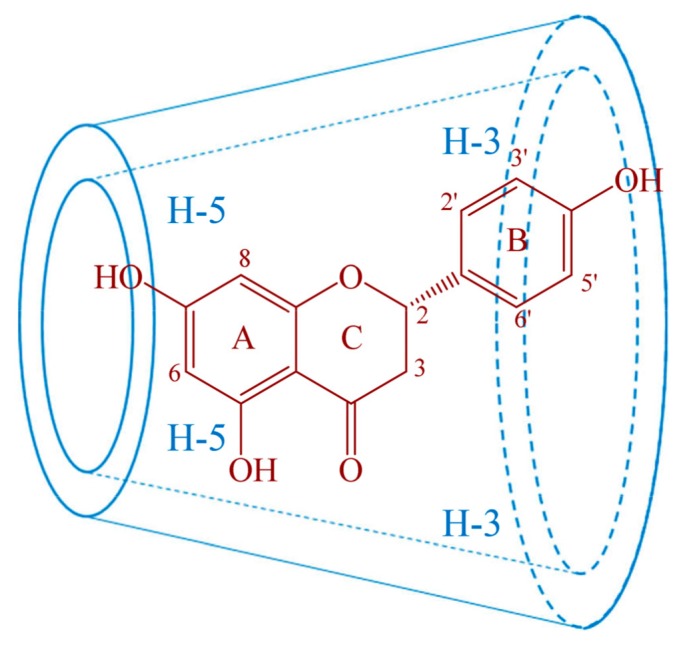
The possible inclusion mode of naringenin with HPβCD.

In order to further confirm the complexation mode of naringenin-HPβCD inclusion, 2D ROESY spectroscopy was utilized. It is able to reveal the spatial relationships between protons of guest and host molecules of cyclodextrin inclusions. Two protons that are closely located in a space can produce a nuclear Overhauser effect (NOE), presenting as a cross-peak between protons from two molecules in the 2D ROESY spectrum [28]. The ROESY spectra of naringenin-HPβCD inclusion (Figure 5) showed cross-peaks between H-6 and H-8, H-2′ or H-6′ and H-3′ or H-5′ protons of naringenin and H-3 and H-5 protons of HPβCD. Consistent with the observations of ^1^HNMR, the results of 2D ROESY indicated that the entire naringenin molecule was involved in the cavity of HPβCD. The possible inclusion mode of naringenin with HPβCD is illustrated in Figure 6.

### 2.5. Permeation Study

In order to predict the permeation characteristics of naringenin and its cyclodextrin inclusion, Calu-3 cells were used as a pulmonary epithelial cell model, which can form tightly confluent monolayers with suitable permeability properties for modeling the airway epithelial barrier, resulting in a wide range of applications in permeation studies [29,30]. Furthermore, compared to submersed-liquid culture, Calu-3 cells cultured in air-liquid conditions can provide enhanced ciliogenesis, increased mucus secretion, and tighter junction formation, which is similar to the environment in the respiratory tract [31,32].

The transepithelial electrical resistance (TEER) values of Calu-3 cells are shown in Table 2. After being seeded on the Transwell filter plates, the TEER values of Calu-3 cells increased progressively and reached their plateau at about 580 Ω cm^2^ for 12–14 days. These results were consistent with other studies, in which the TEER values of Calu-3 cells grown in air-liquid culture were 350–1200 Ω cm^2^ [22,33], indicating that the cells already form tight confluent layers for permeation study. There was no significant difference between the TEER values at the beginning (663 ± 45 Ω) and at the end of the study (657 ± 50 Ω). Thus, the cell layers remained intact during the permeation study.

**Figure 7:**
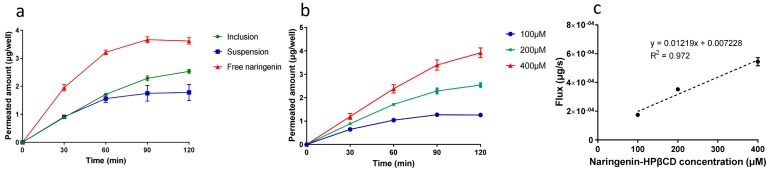
Cumulative permeated amounts of (**a**) different forms of naringenin and (**b**) different concentration of naringenin inclusion (**b**), (**c**) naringenin flux of different concentration of naringenin inclusion. (mean ± SD, *n* = 4).

In the comparison of the permeation characteristics of three different forms of naringenin, naringenin solution showed the highest permeated amount, which was followed by that of inclusion and suspension (Figure 7a). As presented in Table 3, the permeated amount of naringenin solution was about 1.4 and 2.0 times higher than that of inclusion and suspension, respectively. The absorption of a drug is fundamentally dependent on its hydrophilicity and lipophilicity. Octanol-water partition coefficient (log P), as a parameter of lipophilicity, can be used to predict the affinity of a drug for the cell membranes. It was reported that the log P value of naringenin is 2.60 [34], which means that naringenin can easily transport across the cell membranes. However, prior to transporting across the cell membranes, the drug must dissolve in the apical lining fluid of the cells, which is difficult for naringenin due to its poor solubility and dissolution rate. Complexation with HPβCD could increase the solubility of naringenin, thus providing a higher drug concentration in the apical lining fluid for permeation, which resulted in an increased permeated amount of naringenin compared with the suspension. The permeated naringenin of the inclusion form was lower than that of the solution form, which could be attributed to the permeability of HPβCD. As compounds with large molecular weight and polar character, the permeability coefficients (Papp) values of cyclodextrins on Calu-3 cells measured in a previous study were about 7 × 10^−8^ cm/s [35], which were much lower than that of naringenin (8.17 × 10^−6^) calculated in the present study, and the transport mechanisms of cyclodextrins were passive diffusion. The absorptions of cyclodextrins were also proved in vivo; the systemic bioavailabilities of 66–80% were reported with intratracheal instillation in rabbits [35]. Based on the previous in vitro and in vivo studies, it could be speculated that HPβCD could be absorbed by pulmonary epithelia cells. Therefore, we deduced that two different forms of naringenin existed in the inclusion system for permeation, one was the free naringenin, and the other one was the complexed naringenin. The complexed form of naringenin with lower Papp value may act as a sustained release system for naringenin. As shown in Figure 7a, while the permeated amount of naringenin solution reached the plateau, the naringenin-HPβCD inclusion still showed an increasing trend. Therefore, the application of HPβCD promoted the permeation not by directly increasing the permeability of naringenin, but by increasing the drug concentration in the apical lining fluid of cells and acting as a sustained release system for naringenin.

The transport mechanism of naringenin-HPβCD inclusion was also evaluated by the concentration-dependence study. As shown in Figure 7b, the permeated amount of naringenin was proportional to the inclusion concentration, which could be further demonstrated by the linear relation of the flux values of naringenin in this study (Figure 7c), suggesting that at least in the studied concentration range, the transport of naringenin-HPβCD inclusion was a diffusion-driven process without the participation of membrane transporters. Similar results were observed previously by complexing other drug with HPβCD [23].

### 2.6. Pharmacokinetic Studies

**Figure 8:**
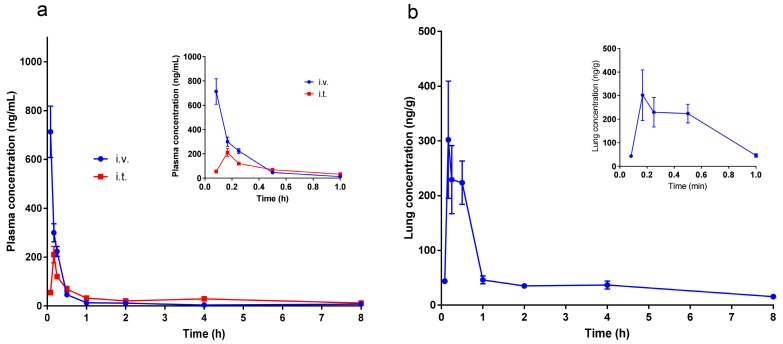
(**a**) Plasmatic pharmacokinetic profile of naringenin inclusion of i.v. and i.t. administration, inset was the magnification of the profile within 0.083~1 h; (**b**) pulmonary pharmacokinetic profile of naringenin inclusion of i.t. administration, inset was the magnification of the profile within 0.083~1 h (mean ± SEM, *n* = 6).

It is reported that naringenin would be metabolized and conjugated during its absorption across the gastrointestinal tract and on circulating through the liver, the major metabolic process is glucuronidation, and most naringenin that exist in plasma are in glucuronidated forms [36]. Previous pharmacokinetic studies demonstrated the low bioavailability of naringenin. After oral administration to rats, the absolute bioavailability of free naringenin was only 3.8%, while it increased to 39.8% after taking the conjugates into account [37]. The tissue distribution study of oral administration showed that most naringenin concentrated in the gastrointestinal tract, followed by kidney, liver, lung, heart, and spleen. Different from the plasma, most naringenin detected in the lungs was in aglycone form, which may attribute to the increased polarity and molar weight of the conjugates [16]. Therefore, the naringenin concentration in lungs was low after oral administration. In the present work, we studied the plasmatic and pulmonary profiles of naringenin after pulmonary delivery. The concentration-time curves and the pharmacokinetic parameters are present in Figure 8 and Table 4. With the rapid absorption of pulmonary delivery, the T_max_ of intratracheal instillation (i.t.) group was 0.181 h, which was slightly longer than that of the intravenous group (i.v.) (0.083 h). The absolute bioavailability of the intratracheal instillation group was 87% when the naringenin conjugates were taken into account, indicating a remarkable enhancement compared with that of oral administration (39.8%) [37]. Consistent with the systematic profile, the T_max_ of lung tissue profile was 0.181 h. The C_max_ and the area under the concentration-time curve AUC_(0-t)_ were 302.09 ng/g and 378.21 ng/g*h, respectively. Compared with the previous tissue distribution study of oral administration of naringenin glucoside at 42 mg/kg dose [17], in which the C_max_ and AUC_(0-t)_ of lung were 54.76 ng/g and 251.34 ng/g*h, pulmonary delivery presented higher C_max_ and AUC_(0-t)_ values with much a lower dosage. Intratracheal instillation can provide prediction of the pharmacokinetic profiles of pulmonary delivery, however, it cannot completely mimic the clinical delivery, in which the inhalation solutions would be deliver as aerosols with nebulizers and inhaled from the nose or mouth. Therefore, further investigations are needed to clearly illustrate the pharmacokinetic performance of pulmonary delivery of inhalation solutions.

Compared with other formulation strategies of naringenin in the literatures, such as self-nanoemulsifying drug delivery system, solid dispersions and nanoparticles, the composition of our formulation is simple with HPβCD as the only excipient. HPβCD is used extensively as a safe and effective solubilizing excipient in various delivery routes. It was reported that cyclodextrins were introduced to the formulation of naringenin for oral and ocular delivery [12,15]. In our study, the feasibility of preparing a naringenin-HPβCD inclusion for pulmonary delivery was evaluated. With the superiority of pulmonary delivery, the absolute bioavailability is more than twice that of oral administration. In addition, while the T_max_ of lung tissue in oral naringenin formulation was 0.5 h [14], the one of pulmonary delivery was 0.183 h, which is crucial for the treatment of respiratory diseases. The ratio of HPβCD used in our formulation was relatively high; therefore, in future study, the preparation and formulation of the naringenin-HPβCD inclusion would be optimized to further reduce the amount of HPβCD.

## 3. Materials and Methods

### 3.1. Materials

Naringenin (purity: 95%) was purchased from Yanhao Biotechnology Co., Ltd. (Xi’an, China). HPβCD (MW 1374) was purchased from Binzhou Zhiyuan Biotechnology Co., Ltd. (Binzhou, Shandong, China). Naringenin, isoquercitrin reference standards, and β-glucuronidase/sulfatase (Type H-1) were purchased from Sigma-Aldrich (St. Louis, MO, USA). Mass spectrometry (MS) grade methanol and formic acid were purchased from Fisher Scientific (Fair Lawn, NJ, USA). High-performance liquid chromatography (HPLC) grade methanol and ethyl acetate were purchased from Honeywell B&J (Albany, NY, USA). Water was prepared using a Milli-Q purification system (Millipore, MA, USA)

Dulbecco’s modified Eagle’s medium/F12 (DMEM/F12), MEM non-essential amino acid (MEM NEAA), and fetal bovine serum (FBS) were purchased from Gibco (Grand Island, NY, USA). Penicillin-streptomycin and Hank’s balanced salt solution (HBSS) were purchased from Hyclone (South Logan, UT, USA). Dimethyl sulfoxide (DMSO) was purchased from Sigma-Aldrich (St. Louis, MO, USA).

### 3.2. Phase Solubility Study

An excess amount of naringenin was added to 20 mL of HPβCD solutions of which the concentration range was from 14.29–114.29 mM. The suspensions were stirred in shaker (MaxQ 6000, Thermo Scientific^TM^) at 25 °C for 48 h to reach equilibrium. Naringenin-HPβCD inclusion solution was obtained by filtering the suspensions with 0.45 μm filter. The naringenin concentration was detected by UV spectroscopy after sample dilution. The maximum absorbance of naringenin is 288 nm, while HPβCD shows no absorbance at the same wavelength. Each experiment was carried out in triplicate. The concentration of solubilized naringenin (mM) was plotted against the corresponding concentration of HPβCD (mM). The slope and y-intercept (So) of the linear regression was used to calculate the complexation parameter as follows:

Stability constant, Ks = slope/[So × (1 − slope)], (1)

Complexation efficiency, CE = slope/(1 − slope),(2)

### 3.3. Preparation of Naringenin-HPβCD Inclusion

According to the results of the phase solubility study, the molar ratio of naringenin to HPβCD in saturated condition was 1:5. Considering the stability of the formulation, all naringenin-HPβCD solutions used in present study were prepared with double amounts of HPβCD, and the molar ratio was 1:10. For the subsequent XRD, DSC, IR, and NMR studies, naringenin and HPβCD were added to water and shaken in a flask at room temperature for 12 h, then the inclusion powder was accessed by lyophilization of the filtered naringenin-HPβCD solution. For permeation and pharmacokinetic studies, corresponding concentrations of naringenin-HPβCD solutions were prepared with the same method, while the solvents were replaced with HBSS and saline, respectively.

### 3.4. XRD

XRD experiments of naringenin, HPβCD, and naringenin-HPβCD inclusion were performed on an X-ray diffractometer (Empyrean, PANalytical Corporation, Almelo, Netherlands). A copper anode providing CuKα radiation at 40 kV and 40 mA was adopted. The recorded range of 2θ was from 3° to 40°.

### 3.5. DSC

DSC experiments of naringenin, HPβCD, and naringenin-HPβCD inclusion were performed on DSC-204 (Netzsch, Germany) under 70 mL/min of nitrogen flow. Signals were recorded within 30–300 °C at a heating rate of 10 °C/min.

### 3.6. HNMR and 2D ROESY

^1^HNMR and 2D ROESY experiments were performed on a Bruker AVANCE III 600 spectrometer (Billerica, MA, USA). 2D ROESY spectrum was recorded with a mixing time of 300 ms, and 16 scans were collected for each of the 256 experiments. In all experiments, the probe temperature was maintained at 298 K and standard 5 mm NMR tubes were used. In ^1^HNMR experiments, all samples were dissolved in DMSO-*d*_6_, while in the 2D ROESY experiment, naringenin-HPβCD inclusion was dissolved in D_2_O.

### 3.7. Permeation Study of Naringenin- HPβCD Inclusion

#### 3.7.1. Cell Culture

Calu-3 cells were gifts from Dr. Wing-Hung Ko at The Chinese University of Hong Kong, Hong Kong, China. The cells were cultured with DMEM/F12 supplemented with 10% fetal bovine serum, 1% MEM NEAA, and 100 U/mL penicillin-streptomycin solution at 37 °C in a humidified atmosphere of 5% (*v/v*) CO_2_. The cultured medium was changed every 2–3 days and the cells were passaged weekly before seeding. To obtain confluent cell monolayers, Calu-3 cells were seeded on Transwell^®^ filter plate (12 mm diameter inserts, 0.4 μm pore size, Corning, NY, USA) at a density of 5 × 10^5^ cells/well with 500 μL medium in the apical chamber and 1500 μL medium in the basolateral chamber. After 2 days, the apical medium was removed to create an air-liquid interface culture, and the medium of basolateral chamber was changed to 1000 μL. The medium was changed every 2 days.

#### 3.7.2. TEER Values Measurement

The TEER values were measured every 2–4 days with Millicell^®^-ERS-2 (Merck Milipore, Billerica, MA, USA). When measuring TEER values, 500 and 1500 μL medium were added to the apical and basolateral chamber, respectively. Prior to the measurement, the cells were equilibrated for 30 min in the incubator. After 12–14 days, the cell layers with stable TEER values (>500 Ω) were ready for the permeation study. To monitor the integrity of the cell layer, TEER values were also determined before and after the permeation study by replacing the cultured medium with HBSS.

#### 3.7.3. Permeation Study

To access the influence of HPβCD on the permeation of naringenin, the permeation characteristics of three different forms of naringenin (solution, suspension, and naringenin-HPβCD inclusion) were evaluated at the same dose (200 μM). Naringenin solution was prepared by dissolving naringenin in DMSO due to its poor solubility, and then the stock solution was diluted with HBSS to reach the final concentration (the concentration of DMSO in this solution was 0.1% (*v/v*)). Naringenin suspension was prepared by adding the corresponding amount of naringenin powder in HBSS, and in order to evenly disperse the naringenin powder, the suspension was ultrasound for 1 h. For the concentration-dependent study, 100, 200, and 400 μM naringenin-HPβCD inclusions were prepared according to the method mentioned in Section 2.3. Both the concentrations of naringenin and naringenin-HPβCD inclusion applied in this study exhibited no cytotoxicity with CellTiter 96^®^ AQueous One Solution Cell Proliferation Assay (MTS) in preliminary studies. Before the permeation study, the cell layers were washed twice by 500 μL HBSS, then the apical chamber and basolateral chamber of each well were filled with 500 μL and 1500 μL HBSS, respectively, for TEER determination. After that, the HBSS in the apical chamber was discarded, and the volume of basolateral chamber was changed to 1000 μL, then 150 μL drugs were pipetted onto the cell layers. The transwell plates were agitated by using an orbital shaker at 200 rpm stirring rate and at 37 °C during the entire exposure time. Then, 120 μL samples from the basolateral chamber were taken at 0, 30, 60, 90, and 120 min and replaced with pre-warmed HBSS buffer; the naringenin concentration of sample was assayed by HPLC. TEER values were measured after the permeation study to investigate the influence on cell layers.

Papp value was calculated according to the following equation, where dQ/dt is the flux across the cell monolayer (μg/s), A is the surface of the monolayer (cm^2^), and C is the initial concentration in the apical chamber (μg/mL)

P_app_ = dQ/(dt × A × C), (3)

#### 3.7.4. HPLC Assay

The concentration of naringenin was quantified by Ultimate 3000 DGLG HPLC (Dionex, Waltham, MA, USA) equipped with LPG-3400SD pump, WPS-3000SL sampler, TCC3000-RS column oven, and photo-diode-array-detector (DAD). The analytical column used was Elite Hypersil ODS2 (4.6 × 250 mm). The mobile phase consisted of methanol and water in a 55:45 ratio. The flow rate was 1 mL/min, and the column temperature was 25 ℃. The maximum absorbance of naringenin was 288 nm. Linearity was proven in the range of 0.5 to 20 μg/mL. The validation parameters of the methodology of HPLC assay is provided in the Supplementary Materials.

### 3.8. Pharmacokinetic Study

#### 3.8.1. Animals

Male and female Sprague-Dawley (SD) rats weighing 180–220 g (9 weeks-old) were supplied by Guangdong Medical Laboratory Animal Center (Guangzhou, China). Animals were acclimated at least 3 days before the pharmacokinetic study to environment with 20–25 ℃ and 55 ± 15 % humidity, 12 h light/dark cycle was applied. Food and water were provided ad libitum. The animals were fasted overnight with free access to water before the experiment. All experimental procedures complied with the National Institutes of Health Guide for the Care and Use of Laboratory Animals (NIH Publications No. 8023, revised 1978), and were approved by the Animal Ethics Committee of the School of Life Sciences in Sun Yat-sen University (approval number: 180613 and 180614, date: 2018/06/08).

#### 3.8.2. Administration Protocol

For the intratracheal instillation study, 48 SD rats (half male and half female) were randomly divided into 8 groups corresponding to the predetermined sample collection time points (0.083, 0.167, 0.25, 0.5, 1, 2, 4, 8 h). Animals were intraperitoneally injected with 10% chloral hydrate (*w*/*v*, g/mL) for anesthesia, and then a volume of 50 μL/100 g of naringenin-HPβCD inclusion was given to the rats. The dose was 400 μg/kg. After intratracheal instillation administration, the whole blood was collected from the retro orbital at the aforementioned time points. Animals were sacrificed by cervical dislocation, the lungs were excised by surgical resection, and the visible bronchi were removed. For intravenous administration, 6 SD rats (half male and half female) were randomly selected and administrated with the same dose intravenously. Blood samples were collected at the same time points as mentioned before. Plasma samples were obtained by immediate centrifugation at 4000 r/min centrifugation for 10 min at 4 °C. Both plasma samples and lung tissues were stored at −80 °C before assay. The samples of pharmacokinetic study were quantified by liquid chromatography-triple quadrupole mass spectrometry (LC-MS/MS, Agilent Technology, Santa Clara, CA, USA). The sample preparation and quantitative methods are referred to in our previous studies [17,38].

## 4. Conclusions

This study showed that complexation with HPβCD was able to increase the solubility of naringenin effectively. ^1^HNMR and 2D ROESY studies illustrated that the entire naringenin molecule was involved in the cavity of HPβCD to form a 1:1 inclusion. The application of HPβCD could not only increase the naringenin concentration in the epithelium-lining fluid (ELF) of pulmonary epithelial cells, but also act as a sustained release system. Furthermore, pharmacokinetic study showed that pulmonary delivery of naringenin-HPβCD inclusion provided rapid response and higher local concentration of naringenin, which is critical for improving the therapeutic effects for respiratory diseases.

## Figures and Tables

**Table 1:** The chemical shift of naringenin and HPβCD in free and inclusion state.

Compound	Proton	Chemical Shift δ (ppm)		Δδ (ppm)
		δ (Free)	δ (Inclusion)	
Naringenin	2	5.438	5.427	−0.011
	6 or 8	5.878	5.857	−0.021
	3′ or 5′	6.789	6.785	−0.004
	2′ or 6′	7.312	7.308	−0.004
HPβCD	H-3	3.736	3.755	0.019
	H-5	3.468	3.470	0.002

**Table 2:** TEER values of Calu-3 cells.

Day	4	8	10	12	14
TEER (Ω)	212 ± 25	462 ± 33	510 ± 70	576 ± 30	581 ± 31

**Table 3:** Permeated amounts and flux values of different forms of naringenin.

Form	Permeated Amount (μg/well)	Flux (μg/s × 10^−4^)
Naringenin solution	3.61 ± 0.11	5.03 ± 0.16
Naringenin inclusion	2.53 ± 0.06	3.52 ± 0.09
Naringenin suspension	1.78 ± 0.30	2.47 ± 0.40

**Table 4:** Plasmatic pharmacokinetic parameter of naringenin inclusion of i.v. and i.t. administration.

Parameter	IV	IT
AUC0-t (μg/L∗h)	268.20 ± 85.62	233.60 ± 37.47
AUC(0-∞) (μg/L∗h)	273.00 ± 84.46	291.15 ± 77.18
Tmax (h)	0.083	0.181 ± 0.034
Cmax (μg/L)	713.12 ± 258.564	217.86 ± 66.35
t_1/2_ (h)	2.25 ± 0.90	4.15 ± 2.13
Clearance (L/h/kg)	1.59 ± 0.51	1.46 ± 0.38
Bioavailability (%)	87 ^1^	

^1^ The absolute bioavailability of 87% was calculated by taking the naringenin conjugates into account.

## Supplementary Materials

The supplementary materials are available online.
