# Supplementary material for: Characterization, in Vitro and in Vivo Evaluation of Naringenin-Hydroxypropyl-β-Cyclodextrin Inclusion for Pulmonary Delivery
Source: Molecules. 2020 Jan 28;25(3):554. doi: 10.3390/molecules25030554 (PMC7036785; doi:10.3390/molecules25030554)
Supplement: Supplementary file 1 [file molecules-25-00554-s001.pdf]

Fourier transform infrared spectroscopy (FTIR)

Method: Naringenin, HPβCD and naringenin-HPβCD inclusion were compacted to disk with potassium bromide, respectively. The samples were spectroscopically scanned by FTIR (NICOLET 6700, Thermo Corporation, USA). The spectra range were recorded within 4000– 400 cm^{-1}.

Result: As shown in Figure S1, the FTIR spectrum of naringenin showed small and sharp absorption peaks between 400 and 1600 cm^{-1}, which could be distinguished from that of HPβCD. The spectrum of inclusion was similar with that of HPβCD, in which the absorption peaks of naringenin were masked by those of HPβCD, suggesting that naringenin was encapsulated in HPβCD

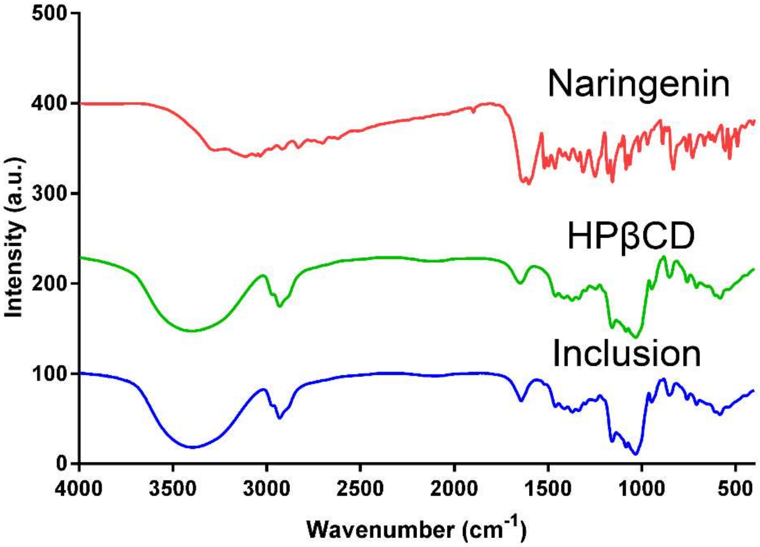

Figure S1: The FTIR spectrum of naringenin, HPβCD and naringenin-HPβCD inclusion

Method validation

Method validation was established according to the Guidelines for drug quality analysis method validation issued by the Chinese Pharmacopoeia Commission in 2015.

Specificity: The specificity of quantitative method was evaluated by comparing chromatograms of blank solvent, vehicle, standard solution of naringenin and sample solution. Sharp and fine peak was obtained for naringenin, and no interference was observed.

Linearity and range: A calibration curve was constructed and fitted by linear least-squares regression analysis to plot the peak area against the concentrations of naringenin. The regression equation of naringenin was $y = 0.6339x - 0.0157$, which obtained over the range 0.4055–81.10 μg/mL with correlation coefficient (r) =1 (shown in Fig S1).

Precision: Precision was assessed by analyses of repeated samples of naringenin-HPβCD inclusion (n = 6), and was expressed by the relative standard deviation (RSD, %). The RSD was 0.85%, which indicated high precision.

Accuracy: Accuracy was assessed by the RSD (%) of recoveries of naringenin, which was determined by comparing the detected concentration to the concentration of the spiked naringenin standard solution at three concentration level, samples prepared in triplicate for each concentration. All the recoveries were over the range 98–102% and the RSD was 1.13%,

showing high accuracy.

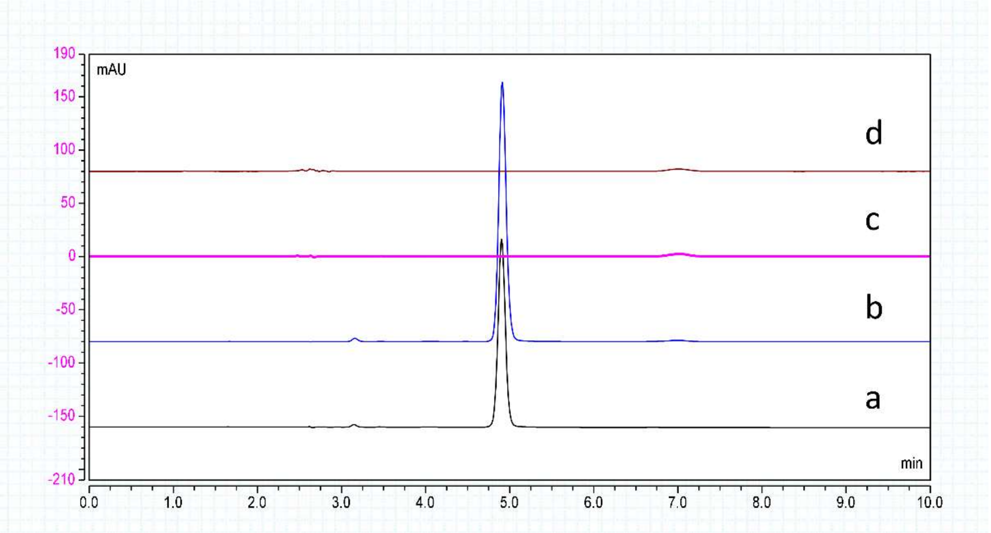

Figure S2 Chromatograms of naringenin sample solution (a), naringenin standard solution (b), blank solvent (c) and vehicle (d)

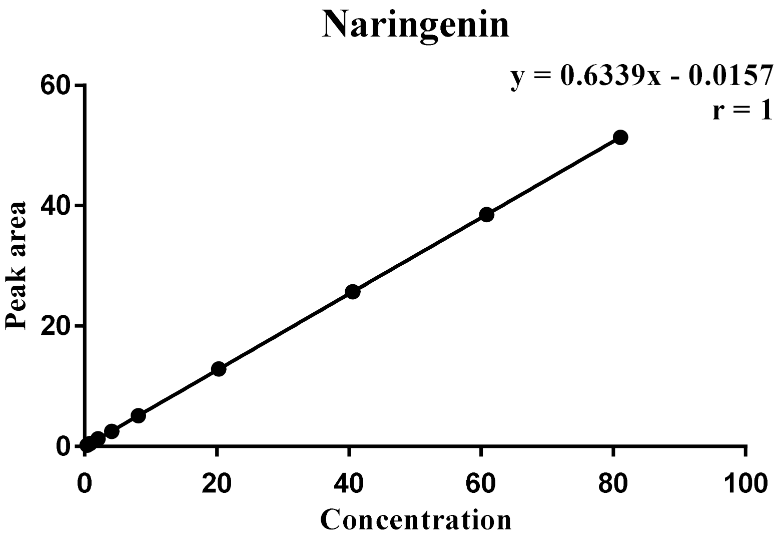

Figure S3 Calibration curve of naringenin.
